# Simultaneous Measurement of Changes in Neutrophil Granulocyte Membrane Potential, Intracellular pH, and Cell Size by Multiparametric Flow Cytometry

**DOI:** 10.3390/biomedicines9111504

**Published:** 2021-10-20

**Authors:** Alexander Elias Paul Stratmann, Lisa Wohlgemuth, Maike Elisabeth Erber, Stefan Bernhard, Stefan Hug, Michael Fauler, Laura Vidoni, Adam Omar Khalaf Mohamed, Bertram Dietrich Thomaß, Frederik Münnich, Laura Stukan, Karl Josef Föhr, Marco Mannes, Markus Stefan Huber-Lang, David Alexander Christian Messerer

**Affiliations:** 1 Institute of Clinical and Experimental Trauma Immunology, University Hospital Ulm, 89081 Ulm, Germany; alexander.stratmann@uni-ulm.de (A.E.P.S.); lisa.wohlgemuth@uni-ulm.de (L.W.); erber.maike@gmail.com (M.E.E.); stefan.bernhard@uni-ulm.de (S.B.); stefan.hug@uni-ulm.de (S.H.); vidoni.laura95@web.de (L.V.); adam.mohamed@uni-ulm.de (A.O.K.M.); bertram.thomass@uni-ulm.de (B.D.T.); frederik.muennich@uni-ulm.de (F.M.); laura.stukan@uni-ulm.de (L.S.); marco.mannes@uni-ulm.de (M.M.); markus.huber-lang@uniklinik-ulm.de (M.S.H.-L.); 2 Institute of General Physiology, Ulm University, 89081 Ulm, Germany; michael.fauler@uni-ulm.de; 3 Department of Anesthesiology and Intensive Care Medicine, University Hospital Ulm, 89081 Ulm, Germany; karl.foehr@uniklinik-ulm.de

**Keywords:** neutrophils, innate immunity, membrane potential, forward scatter, intracellular pH, flow cytometry, lipopolysaccharide

## Abstract

Neutrophils provide rapid and efficient defense mechanisms against invading pathogens. Upon stimulation with proinflammatory mediators, including complement factors and bacterial peptides, neutrophils respond with changes in their membrane potential, intracellular pH, and cellular size. This study provides an approach to quantify these important changes simultaneously using multiparametric flow cytometry, thereby revealing a typical sequence of neutrophil activation consisting of depolarization, alkalization, and increase in cellular size. Additionally, the time resolution of the flow cytometric measurement is improved in order to allow changes that occur within seconds to be monitored, and thus to enhance the kinetic analysis of the neutrophil response. The method is appropriate for the reliable semiquantitative detection of small variations with respect to an increase, no change, and decrease in those parameters as demonstrated by the screening of various proinflammatory mediators. As a translational outlook, the findings are put into context in inflammatory conditions in vitro as well as in a clinically relevant whole blood model of endotoxemia. Taken together, the multiparametric analysis of neutrophil responsiveness regarding depolarization, alkalization, and changes in cellular size may contribute to a better understanding of neutrophils in health and disease, thus potentially yielding innovative mechanistic insights and possible novel diagnostic and/or prognostic approaches.

## 1. Introduction

Neutrophil granulocytes (neutrophils) represent quantitatively the most relevant cell type of the innate immunity. Together with humoral defense mechanisms, such as the complement system, neutrophils serve as a vanguard against the pathogen invasion, thereby forming the “first line of defense” [1,2,3,4]. Neutrophils can be recruited and activated by many proinflammatory factors, for example, by cleavage products of the complement system such as complement factor 5a (C5a), interleukins, and/or microbe-associated molecular patterns (MAMPs), for example, lipopolysaccharide (LPS) [1,4,5]. Upon activation, neutrophils advance to the site of inflammation by chemotaxis, where they exert several typical physiological functions to combat invading pathogens, including the generation of reactive oxygen species (ROS), phagocytosis, and the formation of neutrophil extracellular traps [1,2,4,6]. In the context of systemic inflammation, such as after severe trauma or during sepsis, neutrophils are extensively stimulated [3,7,8,9]. In this regard, excessive neutrophil activity contributes to organ dysfunction and ultimately, mortality [3,4,10]. Moreover, prolonged inflammation may result in paralysis of the neutrophils’ activities, thus impairing the host’s capacity to effectively clear pathogens and resolve inflammation [9,11,12,13].

Neutrophil activation by the above-mentioned chemoattractants typically induces a certain response pattern, including depolarization of the membrane potential (MP), alkalization of the intracellular pH (pH_i_), and a change in cellular shape as reported by our group [4,14,15,16,17,18] and many others [19,20,21,22,23,24]. These distinct alterations are closely associated with crucial neutrophil functions. For example, cellular depolarization likely reflects the activation of the NADPH oxidase (NOX), which generates superoxide anions as an important ROS to kill pathogens [6,20,25,26,27,28,29]. It is of note that patients with defects in NOX activity respond to stimulation with considerably reduced depolarization [28,30]. Neutrophil alkalization is largely mediated by the activity of the sodium-proton-exchanger 1 and of the voltage-sensitive proton channel 1 [4,17,18,31,32]. These variations in pH_i_ influence neutrophil activity. For example, alkalization increases glucose metabolism and lactate generation [15]. Furthermore, changes in the neutrophil pH_i_ are involved in chemotactic activity and apoptosis [21,33,34,35]. Changes in neutrophil cell shape are required for neutrophil extravasation as well as their chemotactic activity, which are associated with actin cytoskeleton polymerization [12,16,36].

To advance the understanding of neutrophil biology in health and disease, changes in their MP, pH_i_, or cell size are of special interest. Consequently, it appears that, particularly for the MP, this may be a domain for the application of standard electrophysiological methods. Indeed, the patch-clamp technique has been successfully applied to register membrane currents of neutrophils. It allowed an indirect estimation of the NOX activity, which plays a prominent role during the “respiratory burst” of these cells [26,37]. A main advantage of this method is that it provides absolute values with a high time resolution. However, when membrane potentials have to be measured in the current-clamp mode, there are certain limitations. Most prominent here are the extreme variations in the MP between individual cells and the considerable fluctuations within individual cells [38]. Different issues, including incomplete sealing, mechanical stimulation, and alterations of the intracellular milieu due to the intracellular perfusion, have been considered as being responsible for these observations [38]. The most prominent role might be played by the high input resistance of these cells, whereby the activation of single ion channels will be sufficient to change the MP by tens of millivolts [38,39]. To compensate for this variability, the number of analyzed cells needs to be increased. However, this is not compatible with the low throughput obtained from conventional single cell measurements by patch-clamp techniques, even though automatic systems are available, that could partially compensate for this. Regarding pH_i_ and cell size, the patch-clamp technique would only be partly appropriate. Even though there is no possibility to measure the pH_i_ directly, the underlying current through proton channels could be assessed. Similar to the MP, to measure changes in pH_i_, fluorescent dyes are used to circumvent this issue [15,17,18,22,40,41,42]. As a surrogate for cell volume, membrane capacity could be measured by patch-clamp techniques. Taken together, in the past it was not possible to measure all three parameters simultaneously. An alternative to the electrophysiological methods is using fluorescent probes, including SNARF (5-(and-6)-carboxy SNARF™-1, acetoxymethyl ester, acetate, for pH_i_) and DiBAC_4_(3) (bis-(1,3-dibutylbarbituric acid)trimethine oxonol, for MP) [14,17,18].

The use of fluorescent probes is well established for measuring the intracellular parameters of neutrophils, including intracellular calcium and the generation of ROS. Both are incorporated in the “Guideline for the use of Flow Cytometry and Cell Sorting in Immunological Studies” [43]. However, to our knowledge, there is no protocol available regarding the simultaneous measurement of MP and pH_i_ in neutrophils. Nonetheless, changes in the response to chemoattractants of neutrophils in their MP and pH_i_ occur in systemic inflammation [14,17,18,42]. For example, we demonstrated that the chemoattractant-induced depolarization of neutrophils is impaired during porcine hemorrhagic shock [14] but increased on exposure to LPS [18] or in the presence of extracellular acidosis [14,17,18]. Additionally, changes in neutrophil size/shape have been reported during sepsis and in severe injuries [16,23].

Therefore, this study describes and characterizes a multiparametric approach for the simultaneous measurement of changes in neutrophil MP, pH_i_, and cellular size by flow cytometry and applies it to neutrophils exposed to several proinflammatory mediators of interest involved in acute systemic inflammation. Moreover, the simultaneous measurement protocol is employed to generate near-realtime data using flow cytometry, thereby reducing the gap in time resolution compared to patch-clamp. Finally, a translational outlook is provided by applying the presented method to quantify the neutrophil response in a clinically relevant model of endotoxemia.

## 2. Materials and Methods

### 2.1. Ethical Approval and Neutrophil Isolation

Blood was drawn from healthy volunteers after the approval of the Ethics Committee of Ulm University (ethic decision number 459/18) and after obtaining written informed consent from the blood donors. Subjects were healthy males or females aged between 18 and 35 years without any signs of infection or other medical problems and under no medication. Human blood was drawn by peripheral venous puncture as described by the World Health Organization guidelines in phlebotomy [44] in monovettes containing 3.2% sodium citrate (Sarstedt, Nürnbrecht, Germany).

Neutrophils were isolated from human blood using the Ficoll-Paque (GE Healthcare, Uppsala, Sweden) density gradient centrifugation and subsequent dextran sedimentation as described previously [14,15,17,18]. Following hypotonic lysis of the remaining erythrocytes and resuspension in Hank’s balanced salt solution with calcium and magnesium (HBSS, Gibco Thermo Fisher, Darmstadt, Germany) that was titrated to a pH of 7.3, the cell concentration was adjusted to 2 × 10^6^ per milliliter.

### 2.2. Fluorescent Staining

Neutrophils were stained with 50 nM DiBAC_4_(3) (Merck, Darmstadt, Germany, for MP) and 1 µM SNARF (Invitrogen Thermo Fisher, Dreieich, Germany, for pH_i_) in HBSS in a light-protected waterbath at 37 °C for 20 min. Cells were centrifuged (340× *g*, 5 min, room temperature (RT)) and resuspended in Roswell Park Memorial Institute medium with magnesium, calcium, glutamine, bicarbonate, and phenol red (RPMI, Gibco Thermo Fisher) including 50 nM DiBAC_4_(3), and incubated for another 10 min before being measured by flow cytometry.

For experiments with previous exposure to proinflammatory mediators prior to restimulation with phosphate-buffered saline containing calcium and magnesium (PBS, Gibco Thermo Fisher, as control (Ctrl)), N-formyl-methionyl-leucyl-phenylalanine (fMLF, Merck) or C5a (Complement Technology, Tyler, TX, USA), neutrophils were incubated for 60 min with one of the following stimuli in HBSS as indicated: PBS (as Ctrl), 10 µM fMLF, 100 ng/mL C5a, 50 ng/mL granulocyte-macrophage colony-stimulation factor (GM-CSF, Merck, Darmstadt, Germany), 40 ng/mL tumor necrosis factor (TNF, Biolegend, CA, USA), or 100 ng/mL LPS (from Escherichia coli, Merck). At 20 min before the end of the 60 min period, cells were stained as described above. After 60 min, the cells were washed, resuspended in RPMI with the respective stimuli and re-stained with 50 nM DiBAC_4_(3) followed by a resting period of 10 min prior to stimulation with PBS (as Ctrl) or fMLF. All the media/buffers used were previously titrated to pH 7.3.

### 2.3. Stimulation and Flow Cytometric Measurement

Doublets were excluded based on the linearity of the forward scatter area (FSC-A) and height (normally <2%). Neutrophils (polymorphonuclear granulocytes consisting mainly of neutrophils) were identified based on their FSC and side scatter (SSC) properties (Supplement Figure S1). Unstained cells were measured to optimize photomultiplier tubes voltages. Cells stained with either DiBAC_4_(3) or SNARF were used to compensate spillover. For example, the spillover of fluorescein isothiocyanate (FITC) into phycoerythrin (PE) and peridinin-chlorophyll-protein complex (PerCP) was determined to range around 15–20% and 1–5%, respectively. PE and PerCP spillover was not corrected, because this is not necessary because the spillover is also included in the SNARF calibration curves. The spillover of PE and PerCP into FITC was <5%. For each time point, the mean fluorescence intensity (MFI) from at least 5000 cells was recorded. Cells were excited by a blue laser at 488 nm and measured in the respective FITC (530/30 nm, for DiBAC_4_(3)), PE (585/42 nm, for SNARF), and PerCP (670 nm, for SNARF) channel using a CantoII flow cytometer (BD Biosciences, Heidelberg, Germany).

Following the staining period, neutrophils were stimulated with PBS (as Ctrl) or with one of the listed inflammatory mediators: 10 µM fMLF, 100 ng/mL C5a, 100 nM leukotriene B4 (LTB4, Cayman Chemical, Ann Arbor, MI, USA), 50 ng/mL desmopressin (Cayman Chemical), 50 pg/mL argipressin (Cayman Chemical), 0.2 ng/mL adrenalin (Sanofi-Aventis Deutschland GmbH, Frankfurt am Main, Germany), 1 ng/mL noradrenalin (Sanofi-Aventis Deutschland GmbH), 50 ng/mL GM-CSF, 40 ng/mL TNF, 100 nM angiotensin II (ATII, Merck), or 5 U/mL thrombin (Cayman Chemical). Cells were analyzed for up to 60 min after stimulation as indicated. Between the measurements, the cells were kept in a light-protected water bath at 37 °C.

To analyze near-realtime kinetics, the samples were measured prior to stimulation, followed by continuous data acquisition for 500 s after stimulation (1580 ± 186 neutrophils per second). Data were evaluated with a non-weighted moving average with a window width of one second as described before [18]. To maintain a stable temperature within the 500 s measurement period, the flow cytometry tube was inserted into a temperature-controlled heating unit (TC-124A Handeheld Temperature Controller, 64–1545, Warner Instruments, Holliston, MA, USA).

### 2.4. Membrane Potential Analysis

**Figure 1:**
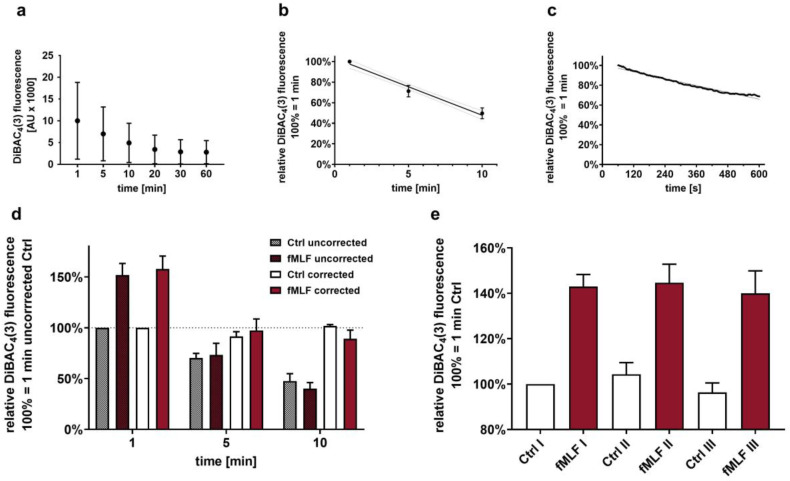
Characterization of the DiBAC_4_(3) fluorescence over time. (**a**) Demonstration of the loss of DiBAC_4_(3) fluorescence in unstimulated neutrophils after a 10 min resting period (*n* = 6). The loss of fluorescence is nearly linear within the first 10 min as evaluated by conventional measurement (*n* = 6) at time points of 1, 5, and 10 min (**b**) as well as by near realtime measurement (representative measurement) (**c**) after a 10 min resting period without further stimulation. (**d**) Illustrates the impact of the correction regarding the loss of DiBAC_4_(3) fluorescence (*n* = 15). (**e**) The measurement of the MP assay has a small coefficient of variation when measuring six tubes consecutively (alternating sequence of independent control and fMLF-stimulated tubes as illustrated) from the same donor normalized to the first tube (*n* = 3).

The reduction in DiBAC_4_(3) fluorescence over time (Figure 1a–c) was corrected for measurements within the first 10 min because control and stimulated cells could not be measured at the same time. This delay between tubes (e.g., unstimulated and stimulated samples) causing a loss of DiBAC_4_(3) fluorescence of approximately 30 s per tube was taken into account for tubes measured within the first 10 min. After 20 min, the relative difference of 30 s compared to 20 min was considered negligible. For this purpose, a linear curve was fitted using the control values of each individual donor of 1, 5, and 10 min. This curve was used to compensate for the loss of DiBAC_4_(3) fluorescence between measurements using the time stamp of each tube and the following equations:

Loss of DiBAC_4_(3) fluorescence:

F_x_ = s × t_x_ + y (1)

F_x_ = Fluorescence at time point x, s = slope, t_x_ = timepoint x [seconds], y = y-intercept.

In a representative time series, the fluorescence at 60, 225, and 505 s were determined to be 3186, 2843, and 2376 AU, respectively. The resulting linear curve fit was F_x_ = −1.804 × tx + 3277 with a correlation coefficient of −0.998.

Correction of the DiBAC_4_(3) fluorescence:

cF = (F_60_/iF_x_) × F (2)

cF = corrected fluorescence, F_60_ = interpolated fluorescence of the control tube measured at 60 s using Equation (1), iF_x_ = interpolated fluorescence of the virtual control tube at x seconds, F = fluorescence of the tube of interest.

A representative calculation is cF = (3168/3114) × 5485 = 5580. In this example, the difference (30 s) between the corrected fluorescence and the measured fluorescence is relatively small, because both tubes of interest were measured in close sequence.

**Figure 2:**
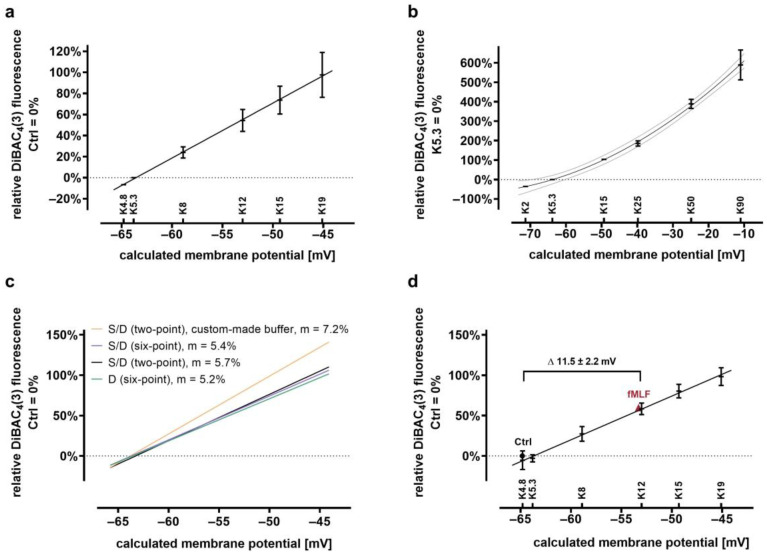
Summary of different calibration curves used to convert changes in DiBAC_4_(3) fluorescence into changes in MP. (**a**) Linear regression curve of DiBAC_4_(3) stained cells. Control cells in DiBAC_4_(3) stained cells in non-modified RPMI were set to 0% (*n* = 3). (**b**) Non-linear regression over a wide range of potassium concentrations (2–90 mM) in custom-made buffer resembling the ion-concentration of conventional RPMI without phenol red and supplemental vitamins. Control cells in custom-made buffer with 5.3 mM potassium were set to 0% (*n* = 3). (**c**) Comparison of different calibration curves. Shown are the calibration curves from (**a**) and linear regression from (**b**) with only potassium concentrations of 5.3 mM and 15 mM included. In addition, a 2-point and a 6-point calibration curve was generated from DiBAC_4_(3)-stained granulocytes in RPMI (*n* = 3, m = slope). (**d**) Example of the application of the generated standard curve (S/D six-point). Cells stimulated with fMLF respond with a depolarization of 11.5 ± 2.2 mV after 1 min compared to unstimulated neutrophils. MP for Ctrl and fMLF-stimulated neutrophils was determined in standard RPMI (*n* = 3).

To demonstrate that DiBAC_4_(3) fluorescence is a valid surrogate of the MP in neutrophils and to quantify changes in the cellular MP, buffers with a varying potassium and sodium content were used. For this purpose, commercially available RPMI as indicated above was mixed with different NaCl/KCl solutions (90% RPMI, 10% NaCl/KCl), resulting in an equimolar exchange of potassium and sodium as indicated in Figure 2a. For example, RPMI contains 138.7 mM sodium and 5.3 mM potassium, therefore, to obtain a modified RPMI with 19 mM potassium, the custom-made NaCl/KCl solution consisted of 2 mM NaCl and 142 mM KCl generated from stock solutions controlled by an osmometer (Gonotec, Berlin, Germany). A second approach with different buffers with ion concentrations similar to those of 4-(2-hydroxyethyl)-1-piperazineethanesulfonic acid(HEPES)-buffered RPMI media was used to test whether certain RPMI-components (e.g., phenol red, vitamins) impact DiBAC_4_(3) fluorescence. For this purpose, a K-free and K-high electrolyte solution (termed “custom-made buffer”) with salt concentrations similar to those of HEPES-buffered RPMI media, but without trace elements, phenol red, nutrients, or vitamin supplements were mixed to achieve a descending dilution series of K^+^ concentrations at equal ion strengths and osmolarities. Concentrations were: 5.6 mM NaH_2_PO_4_, 25 mM HEPES (from buffer with pH 7.5 at RT), 23.8 mM NaHCO_3_^−^, 0.5 mM CaCl_2_, 0.5 mM MgSO_4_, 11.1 mM D-glucose, and 96 mM NaCl for the potassium-free or 6 mM NaCl and 90 mM KCl for the potassium-high solution. Solutions were prepared in a carbogen atmosphere, with pH 7.38 at 37 °C. Osmolarity was adjusted to 280–285 mOsmol/kg by the addition of sucrose (0.25–0.4 g/L).

To convert relative changes of corrected DiBAC_4_(3) fluorescence into changes in MP, a simplified version of the Goldman–Hodgkin–Katz (GHK) equation was used [14,45,46]. Intracellular Na^+^ and K^+^ levels were assumed to be 14 mM and 140 mM, respectively. Relative neutrophil transmembrane permeability was largely potassium-dependent in previous publications [47,48]; therefore, the relative permeability for potassium and sodium was assumed to be 5:95 (for Na^+^:K^+^).

Simplified GHK equation:

U = 61 log × [(P_K_[K^+^]_e_ + P_Na_[Na^+^]_e_)/(P_K_[K^+^]_i_ + P_Na_[Na^+^]_i_) (3)

U = membrane potential, P_K_ = permeability for potassium, P_Na_ = permeability for sodium, [x]_i_ = intracellular concentration of [x], [x]_e_ = extracellular concentration of [x].

Based on Equation (3), a curve fit yielded a slope that expressed the relative change in DiBAC_4_(3) fluorescence in% into change in MP in mV (Figure 2).

### 2.5. Intracellular pH Analysis

To determine the pH_i_, a standard curve was generated using sodium salt from Streptomyces hygroscopicus (nigericin, Cayman Chemical) as described previously [15,49]. This ionophore equilibrates the extra- and intracellular pH milieu. Stained cells were resuspended in RPMI with different extracellular pH levels (5.6; 6.2; 6.8; 7.4; 8.0; 8.6, adjusted by adding either HCl or NaOH) and 10 µM nigericin was added for 30 min. Measured MFI ratios were converted to pH units using the standard curve (Supplement Figure S2).

### 2.6. Analysis of Cellular Size by Flow Cytometry and Electronic Current Exclusion

To determine the cellular size, the FSC-A values generated by polybead polystyrene beads (Polysciences, Warrington, IN, USA) of different diameters (10 µm, 15 µm, 20 µm) were used to establish a calibration curve (Supplement Figure S2). This standard curve directly correlated FSC-A values and cell diameters, thus the cellular size could be calculated. When measuring cell size by flow cytometry, it is important to note that FSC-A values do not directly determine cell volume, but rather give an estimate of altered cell shape, likely representing a change in the length/width ratio [16]. Therefore, neutrophils were stimulated for 10 min and additionally measured by a cell counter working through electronic current exclusion (Cell Counter CASY, OLS OMNI Life Science, Bremen, Germany).

### 2.7. Human Ex Vivo Whole Blood Model

The human ex vivo whole blood model was conducted as previously described [42]. A total of 9 mL blood was drawn into neutral monovettes (Sarstedt) containing heparin (final concentration 0.5 I.U./mL, B. Braun AG, Melsungen, Germany) as an anticoagulant and either PBS (as Ctrl) or LPS. The blood was then transferred into a heparin-coated tubing system (Medtronic, Meerbusch, Germany). The loops containing either blood with PBS (as Ctrl) or 100 ng/mL LPS were then attached to a rotator and circulated at 3 rpm for 1 h at 37 °C. Following the isolation and staining of neutrophils as described above, the cells were stimulated with PBS (as Ctrl) or C5a. To reduce the burden on blood donors, the baseline data (Ctrl-Ctrl and LPS-Ctrl) were shared with another study and have been partially published previously [18].

### 2.8. Data Analysis and Statistics

Data were analyzed using GraphPad Prism 8 (GraphPad Software Inc., San Diego, CA, USA) and Microsoft Excel (version 16.32, Microsoft Corporation, Redmond, Washington, WA, USA). All data are presented as mean ± standard deviation. Comparison of two groups was conducted using the Mann–Whitney test.

## 3. Results

### 3.1. Methodological Aspects

As the first step, the application of DiBAC_4_(3) and SNARF on neutrophils was characterized. The DiBAC_4_(3) fluorescence decreased over the observation period (Figure 1a–d). When this decline is not taken into account, results may vary because control and stimulated cells cannot be measured in parallel by the same flow cytometer. The approach for compensation as described in the method section could address this issue as confirmed in Figure 1e, which shows that the measurement of three different tubes with control or stimulated neutrophils yield comparable results (range coefficient of variation of 3.3–5.3% comparing three measurements from independent sampling tubes from the same donor). To corroborate the fact that DiBAC_4_(3) fluorescence is directly dependent on the MP, and to determine the amount of change in the MP per change in DiBAC_4_(3) fluorescence, various approaches were used to generate calibration curves. Figure 2a demonstrates that the standard RPMI-based approach is capable of reporting hyper- and depolarization, as well as detecting small shifts in MP. Because the standard RPMI-based approach did not allow for an increase in the potassium concentration beyond 19 mM, custom-made buffers resembling ion concentration of RPMI were used to demonstrate that also larger levels of depolarization can be detected by DiBAC_4_(3) fluorescence (Figure 2b). However, beyond approximately −40 mV, an exponential instead of a linear relationship between DiBAC_4_(3) fluorescence and the MP was observed. Subsequently, various DiBAC_4_(3) calibration curves were analyzed (Figure 2c). In this process, it was determined whether the presence of SNARF in addition to DiBAC_4_(3) altered the MP. However, no relevant difference could be detected. Moreover, there was no relevant difference between the results from a two-point- and a six-point-based calibration curve. In this context, it is noteworthy that the slopes (approximately 5–7% with r^2^ = 0.944–0.983 depending on the approach) generated in this series were similar to those produced with slightly different buffers in previous experiments (approximately 6%) [13]. Figure 2d exemplifies the application of the DiBAC_4_(3) calibration curves in analyzing fMLF-stimulated neutrophils. In the course of these experiments, the capability of DiBAC_4_(3) to report large and rapid changes of MP was analyzed using the stimulation of neutrophils with a custom-made buffer increasing the potassium level while lowering the extracellular sodium, resulting in depolarization. After 1 min of increasing the extracellular potassium to 47.7 mM, neutrophil MP was increased by 41.8 ± 2.4 mV in comparison to control cells (*n* = 3, data not shown), which approximately equals the change in MP as calculated by the GHK equation.

The results of the calibration curves of the pH_i_ and cell size are summarized in Supplement Figure S2. The change in cell size indicated by the FSC-A was confirmed by coulter counter measurement. However, the level of the increase varied between the two methods (flow cytometry > coulter counter, Supplement Figure S2) in accordance with previous results [16,17].

### 3.2. Time Course of Depolarization, Alkalization, and Cellular Swelling

**Figure 3:**
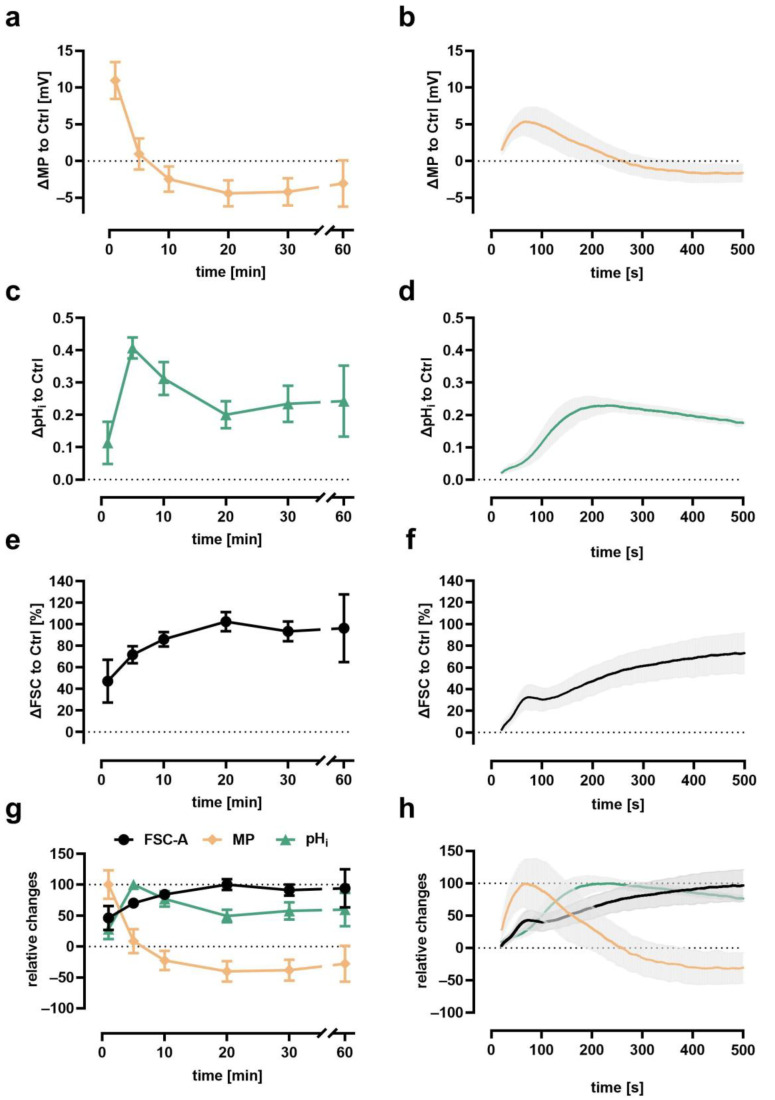
Multiparametric time course of the response of neutrophils after fMLF stimulation by conventional measurement (the same tube measured at multiple time points as indicated, left panel, *n* = 16) or near realtime measurement (the same tube acquired for 500 s after stimulation, right panel, *n* = 6). Synchronous quantification of (**a**,**b**) depolarization, (**c**,**d**) alkalization, and (**e**,**f**) change in cellular size in neutrophils after stimulation with fMLF; (**g**,**h**) summarize the time course of the multiparametric analysis normalized to baseline (0%) and maximal effect (100%). *n* = 5–15, grey areas indicate the standard deviation.

The time course of the fMLF-induced changes in neutrophil MP, pH_i_, and cell size is illustrated in Figure 3. fMLF exposure elicited a rapid depolarization of the MP, peaking around the first two minutes followed by a rapid return to baseline levels with a small hyperpolarization (Figure 3a,b). With a small delay, neutrophil pH_i_ increased with a maximal response at approximately 3 min (Figure 3c,d). In contrast to the depolarization, the alkalization slightly declined and remained elevated during the observation period of 1 h. In parallel, neutrophils responded with an increase in FSC, indicating a change in cellular shape with a maximum at 10 min and a prolonged and sustained increase (Figure 3e,f). The change in MP, pH_i_, and FSC is summarized in Figure 3g,h. It is of note that, in all experiments, MP, pH_i_, and FSC were unimodally distributed among the measured cells, except for a bimodal distribution in pH_i_ and FSC after exposure of neutrophils to LPS in vitro as reported earlier [14,18]. During the measurement, neutrophils stimulated with PBS as Ctrl remained stable (FSC 1 min: 53 ± 22 × 10^3^ AU, 10 min 61 ± 25 × 10^3^ AU, 60 min: 52 ± 28 × 10^3^ AU, *n* = 30, data not shown) at their baseline regarding FSC and pH_i_ (pH_i_ 1 min: 7.18 ± 0.26, 10 min 7.30 ± 0.30, 60 min: 7.12 ± 0.46, *n* = 30, data not shown). Regarding the MP, it cannot be excluded that there is a general drift because of the loss of DiBAC_4_(3) fluorescence, as demonstrated above. The simultaneous measurement of MP, pHi, and FSC enables correlation analysis to be conducted in order to further describe neutrophil biology.

Supplement Figure S3 summarizes the findings for resting and fMLF-stimulated neutrophils. Overall, only small correlations were observed, in addition to a correlation of 0.35–0.48 for SSC and MP. It is of note that comparing neutrophils suspended in RPMI with either 5.3 mM or 15 mM potassium, no significant change in SSC could be noted (SSC of K5.3 190 ± 12 × 10^3^ AU vs. K15 190 ± 13 × 10^3^ AU, *p* > 0.99, *n* = 6, Mann–Whitney test), thus making it unlikely that changes in SSC were accountable for changes in DiBAC_4_(3) fluorescence.

### 3.3. Comparison of the Neutrophil Response Elicited by Different Stimuli

**Figure 4:**
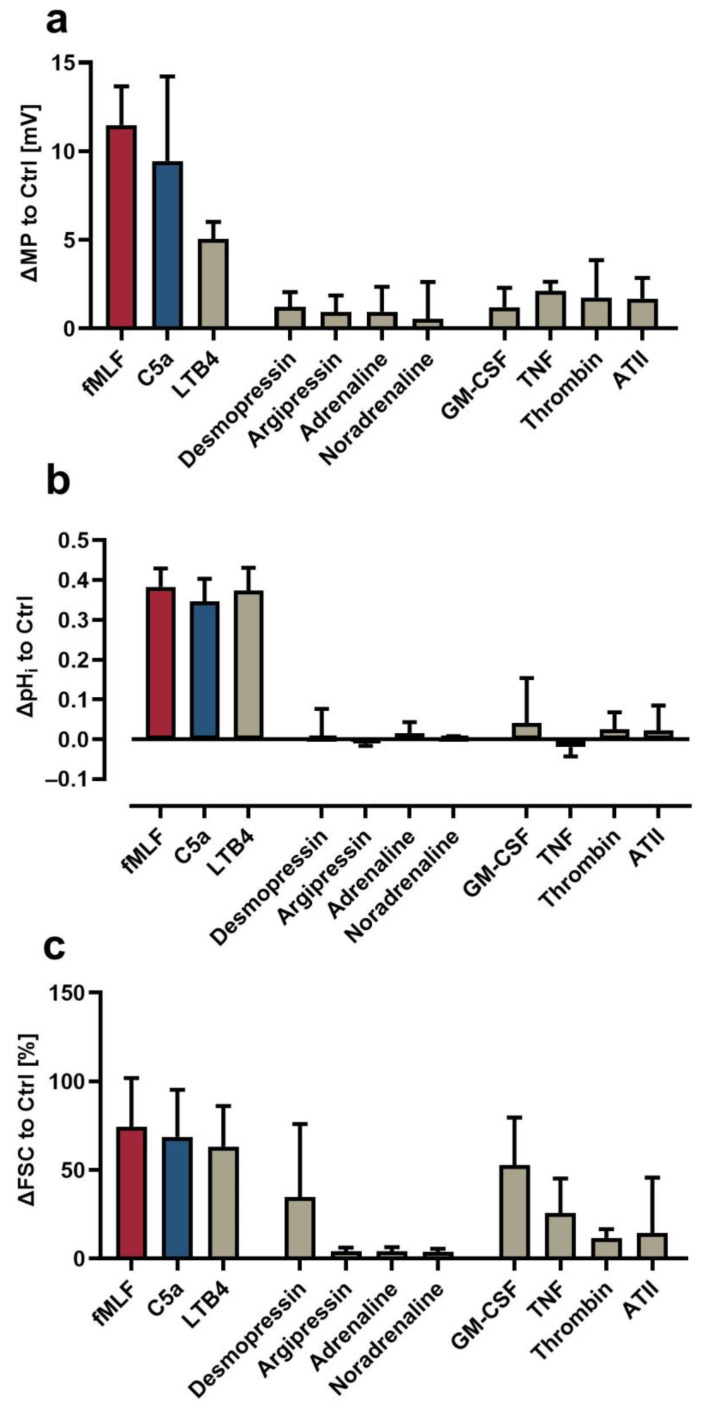
Effects of various sepsis-related mediators including chemoattractants, catecholamines, and other mediators on neutrophils presented at the time of their respective peaks within the first 10 min as determined for fMLF. (**a**) MP after 1 min, (**b**) pH_i_, after 5 min, and (**c**) FSC after 10 min of stimulation (*n* = 5).

The multimodal measurement protocol was subsequently applied to compare the response of neutrophils induced by chemoattractants (fMLF, C5a, LTB4), vasopressors (desmopressin, argipressin, adrenaline, noradrenaline), and other mediators related to inflammation (Figure 4). The chemoattractants fMLF and C5a induced a remarkable change in MP, pH_i_, and FSC, as reported previously [14,15,16]. LTB4 promoted a similar response in neutrophils (Figure 4a). In addition, TNF and GM-CSF increased neutrophil cell size by 25.8 ± 19.5% and 52.8 ± 25.8%, respectively, without triggering depolarization or alkalization (Figure 4b,c). The other screened substances did not largely alter the measured parameters of neutrophil biology.

### 3.4. Exposure to Proinflammatory Stimuli In Vitro Changed the Neutrophil Response to fMLF

**Figure 5:**
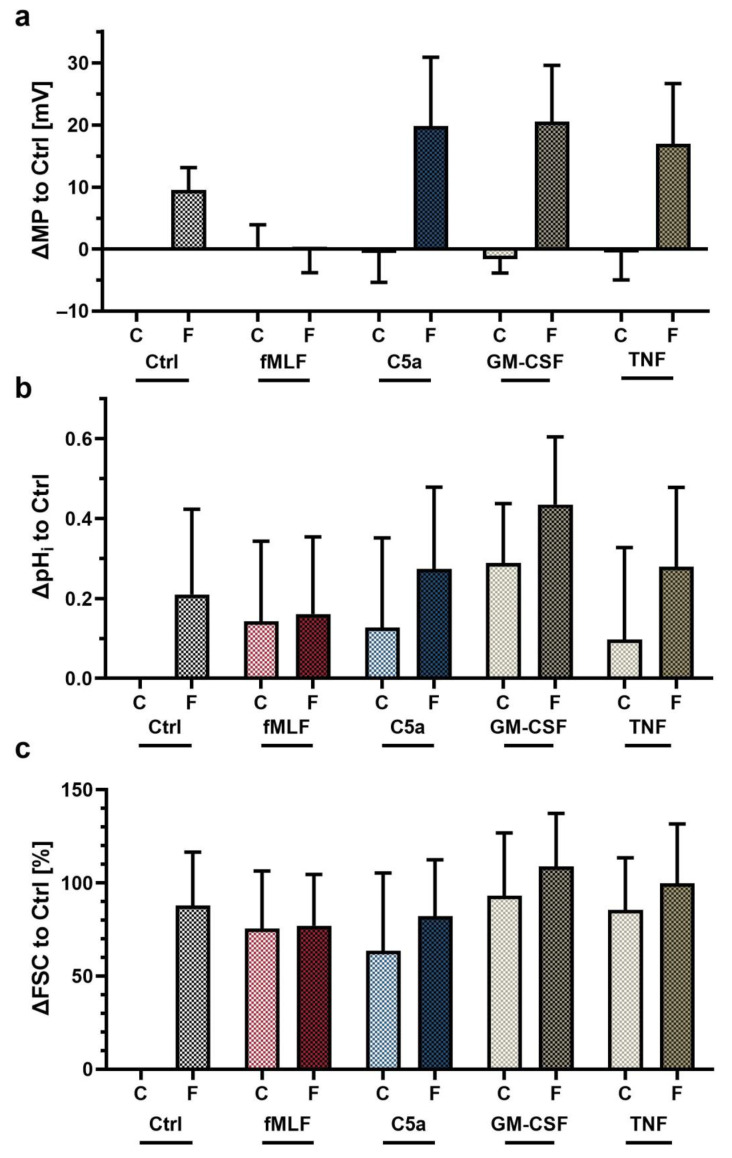
Analysis of the fMLF-induced response in neutrophils after preexposure to various inflammatory mediators for 60 min. Changes in (**a**) MP, (**b**) pH_i_, and (**c**) FSC are altered after preincubation with the indicated substances, staining, centrifugation and resuspension with the same substances followed by stimulation with PBS as Ctrl (C) or fMLF (F) for 1 min (MP), 5 min (pHi) and 10 min (pH_i_), respectively (*n* = 5).

In acute systemic inflammation, neutrophils face several inflammatory stimuli in parallel and/or in a sequence. Therefore, the impact of exposure to various proinflammatory stimuli in vitro on the neutrophil response was investigated. For this purpose, the response of neutrophils to fMLF was analyzed after 60 min pre-exposure to various stimuli, followed by staining of the neutrophils, centrifugation, and resuspension with the same stimuli as indicated. Figure 5 shows that during this procedure, the neutrophil response remained largely intact for depolarization but was diminished regarding the alkalization and cell size as measured by FSC. The fMLF-induced response of cells pre-exposed to fMLF was completely diminished. By contrast, exposure of neutrophils to C5a, GM-CSF, or TNF increased the fMLF-mediated depolarization (Figure 5a). The contact of neutrophils with fMLF, C5a, GM-CSF, or TNF caused sustained pH_i_ and FSC elevation, which was only partially further increased by restimulation with fMLF (Figure 5b,c).

### 3.5. Endotoxemia in Whole Blood Markedly Alters the Neutrophil Response

In the final step, a simplified model of sepsis was applied to briefly characterize the translational value of the measurements. Consquently, neutrophils were exposed to LPS as a typical sepsis-related MAMP followed by stimulation with C5a as a complement cleavage product in response to bacterial invasion. This experimental series was conducted in vitro (using the same method as the experiments in the previous section) and in a clinically relevant ex vivo whole blood model as described in detail before [42]. For example, exposure to LPS in the whole blood model resulted in marked alterations in the neutrophil phenotype regarding activation markers (CD11b, CD14, CD62L, and CD88) and neutrophil activity (ROS generation, phagocytosis) [42].

**Figure 6:**
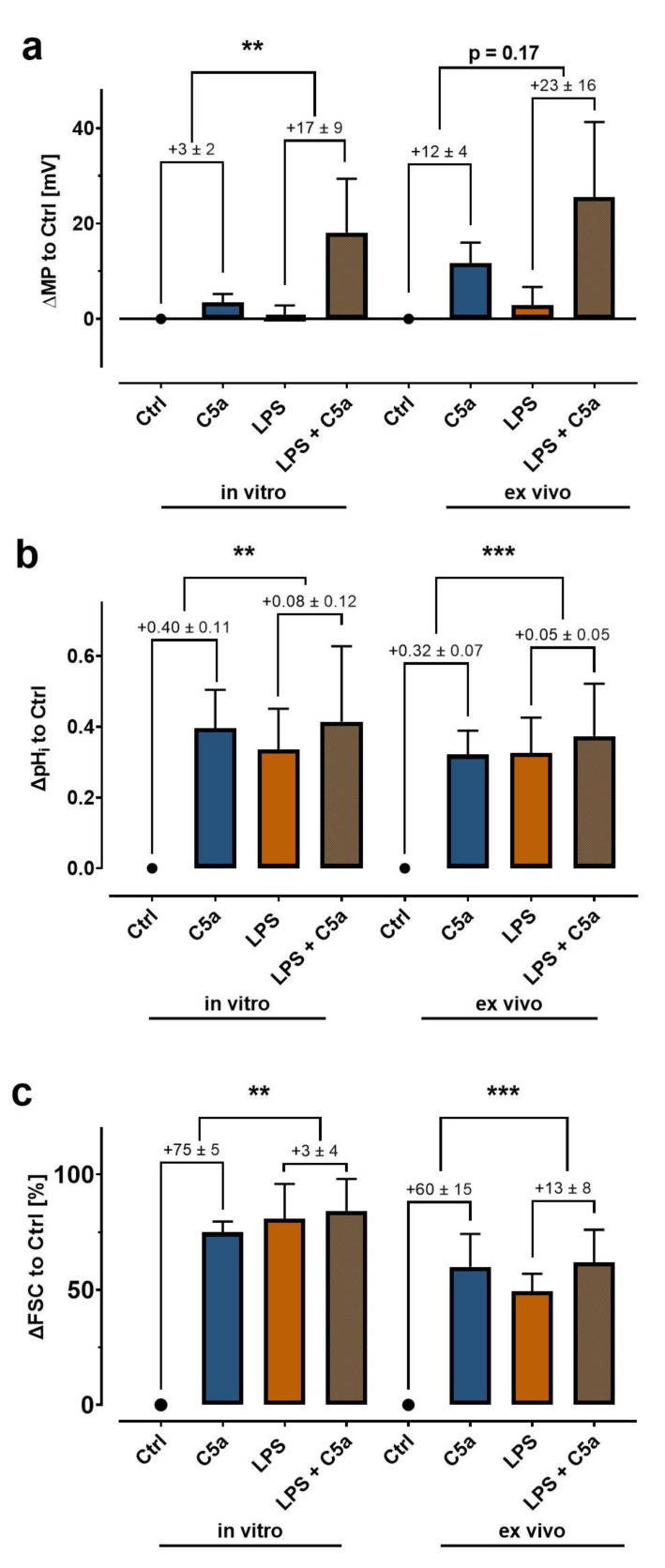
Simplified simulation of sepsis in vitro and in a clinically relevant ex vivo whole blood model. Isolated neutrophils (left) or whole blood (right) was incubated with 100 ng/mL LPS for 60 min followed by subsequent stimulation with 100 ng/mL C5a revealing alterations in the response pattern in (**a**) depolarization, (**b**) intracellular alkalization, and (**c**) changes in cellular size (*n* = 5–8, ** = *p* < 0.01, *** = *p* < 0.001, Mann–Whitney test). Ctrl and LPS data partially overlap from a previous publication [18].

While exposure to LPS did not alter the baseline MP, it augmented C5a-mediated depolarization (Figure 6a). The LPS challenge increased the baseline pH_i_ and FSC. C5a elicited only a reduced increase in pH_i_ and FSC in comparison to neutrophils from blood that was not previousely exposed to LPS (Figure 6b,c). In this context, it is noteworthy that the neutrophils isolated from the whole blood experiment were first exposed to LPS for 60 min and then were subjected to an isolation procedure of approximately 2 h.

## 4. Discussion

Neutrophils provide fast and strong defense capabilities during health and inflammation. Therefore, studying (patho-)physiological patterns of neutrophil activation is of broad interest, including changes in their MP, pH_i_, and cellular shape [1,4,6,23]. However, easy, multimodal, well-standardized, and high-throughput methods with high-temporal resolution to evaluate these parameters are rare [39,43]. This unmet methodological gap is addressed in this paper, accompanied by screening results and a short translational outlook.

The presented approach has certain methodological limitations. MP measurement using a fluorescent dye does not directly report the MP in comparison to a patch-clamp probe. DiBAC_4_(3) is an anion that can cross the plasma membrane; therefore, it accumulates in cells with a positive (or a less negative) MP [46,50,51,52]. As an anionic molecule, DiBAC_4_(3) distributes across the cell membrane according to its Nernst potential. Because transmembrane transport is not facilitated, dye diffusion is slow. Consequently, it is possible that MP changes are underestimated, and reported changes should be assessed on an ordinal scale. However, it is uncertain whether changes in DiBAC_4_(3) fluorescence can mimic every rapid change in MP when this occurs within seconds. Therefore, and because of the results generated in this study, it is possible that the amount of change in MP is underestimated when using DiBAC_4_(3), because this slow-response dye is unable to report a large change in MP when this occurs within seconds [51,52]. Therefore, the authors suggest carefully interpreting the amount of change elicited in the MP as reported by DiBAC_4_(3), and thus rather view it as a surrogate as to whether or not there are changes in the MP. Conventional flow cytometry allows the determination of DiBAC_4_(3) fluorescence at approximately every 30 seconds. This can be somewhat enhanced by continuous measurement, as performed in the present study, thus improving the time resolution to approximately one second. For rapid and/or large changes, it is likely that the response of the fluorescent dyes is unable to precisely serve as a surrogate for MP or pH_i_.

Additionally, to calculate the MP using the GHK equation, several assumptions about ion conductivity and intracellular ion concentrations were required as has been discussed in detail previously [14]. Nevertheless, even if these assumptions should not be entirely correct, only large misassumptions would alter the amount of the change in the MP in a relevant manner [14]. Moreover, the conversion of DiBAC_4_(3) fluorescence into changes in MP does not alter the results of a statistical comparison. Furthermore, using potassium-based calibration curves supports the argument that DiBAC_4_(3) fluorescence is a suitable surrogate for MP. Another important aspect is that, to our knowledge, no reference method (e.g., in direct comparison to neutrophils analyzed by patch-clamp) is available that allows the generation of an absolute reference point using DiBAC_4_(3) in neutrophils. This issue can to some extent be addressed by ionophores such as valinomycin [46,50]. However, these studies yielded varying results from −53 to −102 mV for the resting MP of neutrophils [47,53,54,55]. Regarding the pH_i_, the application of fluorescent indicators is a common but still indirect method for obtaining intracellular proton concentrations [56,57]. The limitations are similar to the measurement of the MP: An alternative approach is the use of ion-selective microelectrodes, which would result in a higher time resolution [57,58,59]. Moreover, changes in MP and pH_i_ were not directly associated with a specific effector function. With respect to changes in FSC, a comparison with results obtained by the coulter counter revealed only small changes in the absolute cell volume. With regard to previous results generated by imaging flow cytometry, changes in FSC are, therefore, more likely to be interpreted as a surrogate of the cellular shape towards an elongated form in relationship with chemoattractant-induced polymerization of the actin cytoskeleton rather than a large increase in total cell volume [16,60]. For all parameters measured by flow cytometry, it has to be considered that once the individual cell has been measured, no replicate measurement using the same cell can be conducted (this issue can be addressed to some extent by cell sorting).

Despite all this, quantifying the response in MP, pH_i_, and FSC by flow cytometry offers multiple advantages. An important aspect is that it enables the analysis of several thousand cells per second. Moreover, the same cell can be simultaneously analyzed regarding its MP, pH_i_, and FSC. Flow cytometers equipped with more lasers may even capitalize further on this benefit, ultimately allowing the analysis of additional parameters such as activation markers and/or calcium signaling in parallel. The evaluation of multiple parameters also reduces the effort required to obtain data for several parameters. In comparison to conventional patch-clamp or the application of microelectrodes, the intracellular milieu of neutrophils remains undisturbed. Therefore, there is no exchange of intracellular ions with a patch pipette or microelectrode solution. Moreover, artificial activation of neutrophils potentially stimulated by contact with the microelectrode can be avoided. Another positive aspect is that samples can be readily maintained at 37 °C, thus more closely mimicking physiological aspects. Finally, flow cytometers are widely available and flow cytometry is easier to master in comparison to the patch-clamp technique, making the method more accessible. This is also beneficial should the neutrophil cell response under certain circumstances and after further evaluations become of diagnostic value, perhaps even as part of an automated point-of-care approach [23,61,62].

It is somewhat unsatisfactory that the fluorescence of DiBAC_4_(3) did not reach a stable level despite multiple variations in the protocol (including varying DiBAC_4_(3) doses, data not shown) within one hour. This might be due to a certain, yet undetermined property of neutrophils. It is noteworthy that DiBAC_4_(3) fluorescence reached a stable plateau in melanoma cells within 10 min [50]. Due to the fact that neutrophils are short-lived cells, and in order to avoid potential unresponsiveness due to long resting periods after incubation, the protocol was optimized to start the measurement after approximately 30 min using the equations as presented in the methods section. Despite the mathematical step needed to compensate for the loss of DiBAC_4_(3) fluorescence, the variation in the methods regarding the MP was <5% when measuring multiple control and fMLF-stimulated cell populations in succession.

The cellular response to various inflammatory mediators with a focus on fMLF and C5a as established stimulants of neutrophils was analyzed. As expected, chemoattractants elicited the greatest response in neutrophils. The results presented in the current study are in agreement with previous findings by our own group and others [14,15,16,18,23,42,63,64]. Following stimulation, neutrophils responded with a clear temporal sequence of depolarization, alkalization, and increase in cell size. The method was able to discriminate peaks in the cellular activity, which, in addition to similar values obtained by single stained control (data not shown), ensures that errors in compensation matrix setup and/or interference of the dyes are unlikely.

The method is capable of reporting changes (decrease, no change, and increase) in MP, pH_i_, and FSC in parallel, which is important because all these parameters may change in pathophysiologically altered neutrophils. For example, neutrophils exposed to fMLF for 60 min were unable to respond with another depolarization to restimulation with fMLF. By contrast and despite C5a as well as fMLF inducing an initial depolarization, neutrophils pre-exposed to C5a responded with an enhanced fMLF-induced depolarization. Additionally, C5a-induced changes in neutrophil pH_i_ and FSC after pre-exposure to LPS and other proinflammatory mediators were reduced, which further exemplifies an alteration in the neutrophil response pattern that can be detected by this method in agreement with results from previous studies [17,18]. This is of special interest and potential translational value, because these alterations have been described earlier in the context of systemic inflammation, as outlined in the introduction [14,17,18,23]. It is tempting to speculate that further improvements and/or applications of the method described in this manuscript may have diagnostic and/or prognostic value in systemic inflammation, including possibly in severe injuries, hemorrhage, or sepsis.

## 5. Conclusions

Changes in neutrophil biology regarding MP, pH_i_, and cell size can be simultaneously evaluated using flow cytometry. The presented method is an effective tool to quantify neutrophil responsiveness in health and during inflammatory conditions. Further studies should improve the methods with a focus on the absolute calibration of the MP. In addition, the dissemination of the presented method may contribute to the generation of novel mechanistic insights, for example, associating changes in MP or pH_i_ to certain cell functions and/or neutrophil phenotypes and to quantify the neutrophil response after stimulation with different inflammatory mediators and/or inhibitors of certain ion transport proteins. Finally, neutrophil responsiveness in these parameters may have translational value as an indicator of immuno(dys-)function in health and acute disease.

## Data Availability

All data will be made available upon reasonable request by emailing the corresponding author.

## Supplementary Materials

The following are available online at https://www.mdpi.com/article/10.3390/biomedicines9111504/s1, Figure S1: Gating strategy, Figure S2: Calibration of the intracellular pH and cell size, Figure S3: Correlation analysis.
