# Supplementary material for: Simultaneous Measurement of Changes in Neutrophil Granulocyte Membrane Potential, Intracellular pH, and Cell Size by Multiparametric Flow Cytometry
Source: Biomedicines. 2021 Oct 20;9(11):1504. doi: 10.3390/biomedicines9111504 (PMC8614908; doi:10.3390/biomedicines9111504)
Supplement: Supplementary file 1 [file biomedicines-09-01504-s001.zip › biomedicines-1411957-supplementary rev2.pdf]

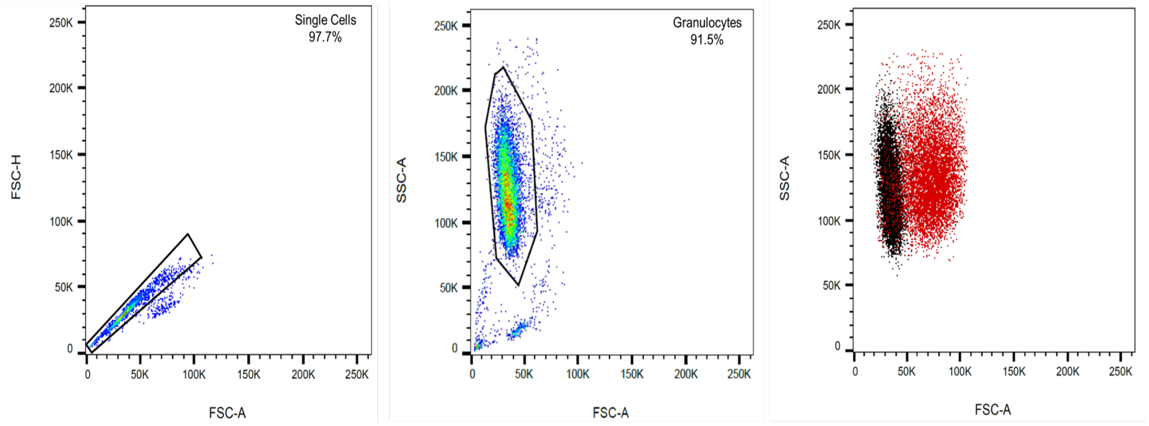


Supplement Figure S1: (a) and (b) summarize the gating strategy to identify granulocytes from a representative donor. (c) Demonstrates the fMLF-induced increase in the FSC-A after 10 min of stimulation with 10 µM fMLF (black = Ctrl, red = fMLF).

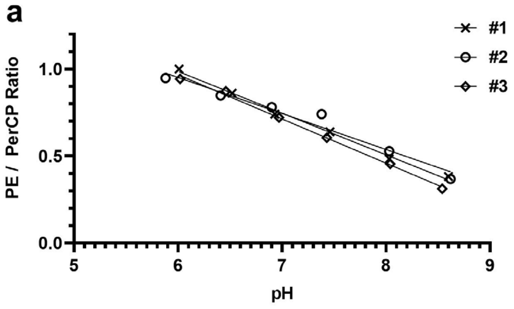


| | #1 | #2 | #3 | mean | SD |
|---|---|---|---|---|---|
| Y-Intercept | 2.425 | 2.193 | 2.492 | 2.370 | 0.128 |
| Slope | −0.240 | −0.207 | −0.254 | −0.234 | 0.020 |
| R² | 0.995 | 0.962 | 0.997 | 0.984 | 0.016 |

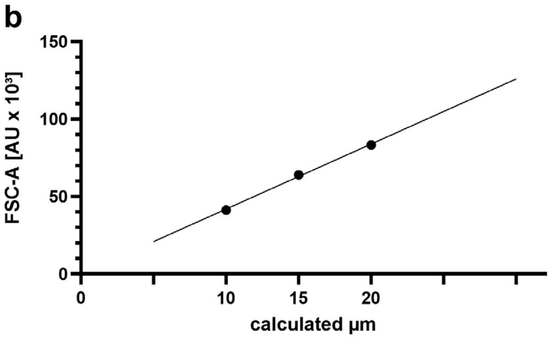


| | #1 | #2 | #3 | mean | SD |
|---|---|---|---|---|---|
| Y-Intercept | 56.7 | 391.5 | −583.2 | −45.0 | 404.4 |
| Slope | 4165 | 4218 | 4215 | 4199 | 24 |
| R² | 0.998 | 0.998 | 0.998 | 0.998 | 0.000 |

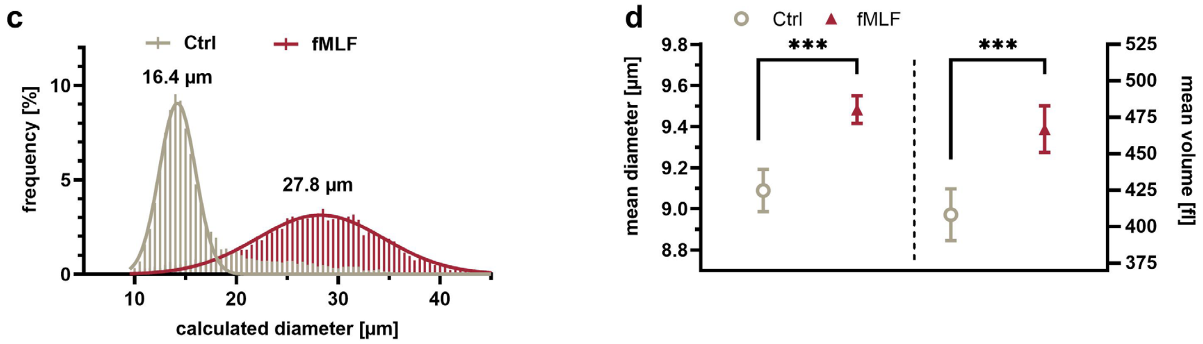


Supplement Figure S2: (a) pH-calibration curves of three donors generated by using the ionophore nigericin converting PE / PerCP ratios of SNARF-stained neutrophils to values of the intracellular pH. (b) Size calibration curves merged of data from three donors using beads with a defined diameter to correlate the FSC-A to cell size in µm (error bars are not visualized due to the small standard deviation). (c) Application of the calibration curve reported in (b) to neutrophils of a representative donor for unstimulated cells or cells stimulated with 10 µM fMLF for 10 min. (d) Analysis of the change in neutrophil diameter (left) and volume (right) after 10 min of stimulation with 10 µM fMLF reveal significant but small changes when analyzed by coulter counter (n = 6, *** = p < 0.001, Mann-Whitney test).

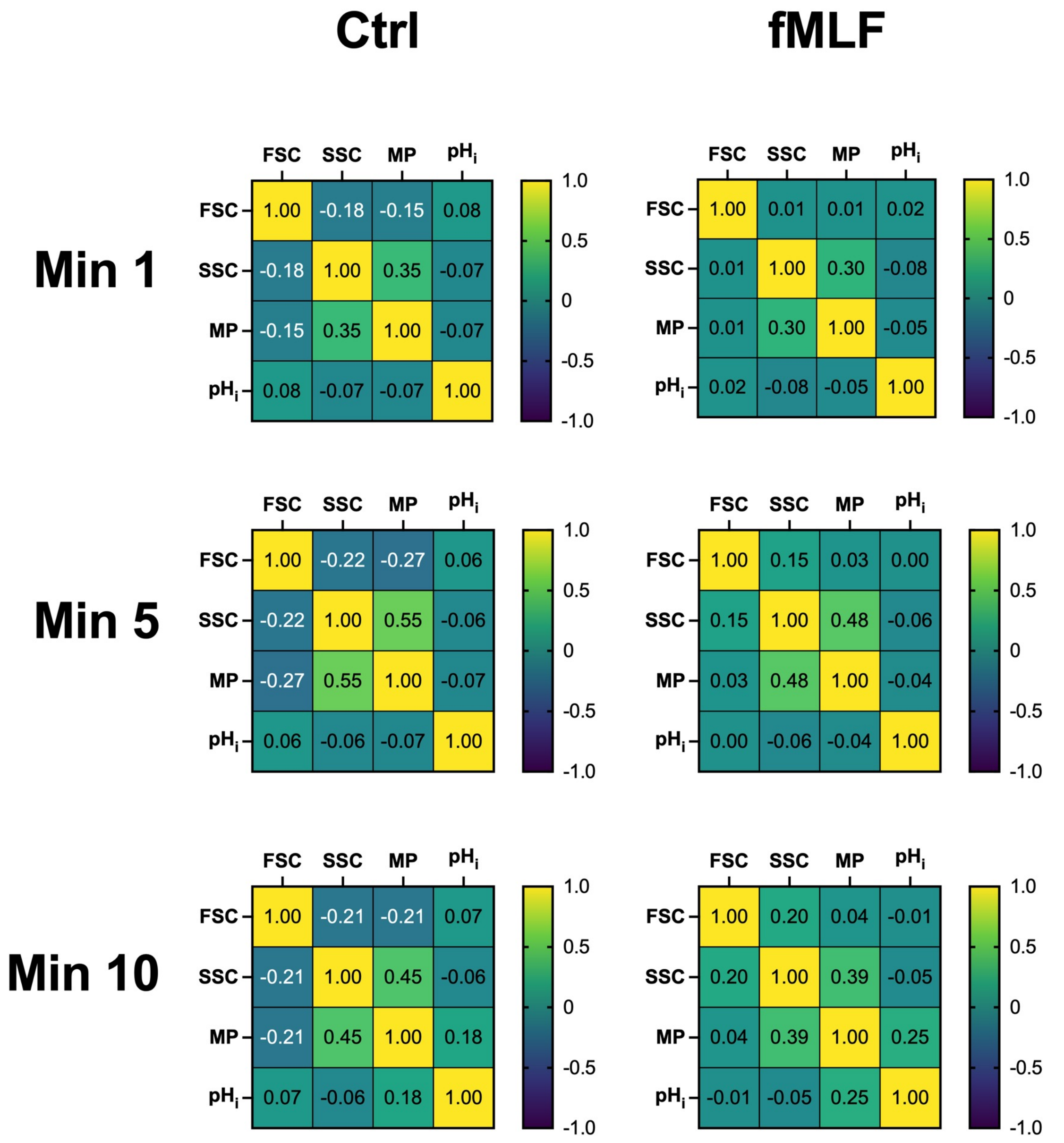


Supplement Figure S3: Mean spearman's rank correlation coefficient from six independent samples analyzing 5000 neutrophils per sample for resting cells (Ctrl) or fMLF-stimulated neutrophils.
